# Poster Session II A326 PATIENTS WITH INEFFECTIVE ESOPHAGEAL MOTILITY HAVE AN INCREASED PREVALENCE OF PATHOLOGIC REFLUX COMPARED TO SYMPTOMATIC PATIENTS WITH NORMAL ESOPHAGEAL MOTILITY

**DOI:** 10.1093/jcag/gwaf042.326

**Published:** 2026-02-13

**Authors:** R Kharfan, K Pokraka, D Y Li, M Buresi, C N Andrews, Y Nasser, M Gupta, M Woo

**Affiliations:** Internal Medicine, University of Alberta, Edmonton, AB, Canada; University of Manitoba, Winnipeg, MB, Canada; Medicine, University of Calgary, Calgary, AB, Canada; Medicine, University of Calgary, Calgary, AB, Canada; Medicine, University of Calgary, Calgary, AB, Canada; Medicine, University of Calgary Cumming School of Medicine, Calgary, AB, Canada; Medicine, University of Calgary, Calgary, AB, Canada; Medicine, University of Calgary, Calgary, AB, Canada

## Abstract

**Background:**

Ineffective esophageal motility (IEM) is the most common abnormality on high-resolution manometry (HRM), yet its clinical significance is uncertain. Previous studies acknowledge the coexistence of IEM and gastroesophageal reflux disease (GERD) but emphasize that symptom correlation is inconsistent and IEM alone is not diagnostic. Clarifying the relationship between IEM, reflux physiology, and symptom burden may improve diagnostic accuracy and management. The Esophageal Symptom Questionnaire (ESQ-30) is a validated tool measuring dysphagia, reflux, and globus through three subscales (ESQ-D, ESQ-R, ESQ-G).

**Aims:**

To compare symptoms and reflux prevalence between patients with IEM and symptomatic patients with normal esophageal motility.

**Methods:**

We retrospectively analyzed HRM reports between July 2022 and October 2024. Patients diagnosed with ineffective esophageal motility (IEM) based on Chicago Classification v4.0 criteria (> 70% ineffective swallows, ≥ 50% failed swallows) were compared to control patients with normal esophageal motility. The symptom profile of patients with IEM was compared to patients with normal motility; in patients who underwent ambulatory pH testing, the prevalence of pathologic reflux (Lyon 2.0 criteria: LA grade B-D esophagitis/Barrett’s esophagus or off-therapy AET [acid exposure time] > 6% or on-therapy AET > 4%), borderline reflux (LA grade A esophagitis or off-therapy AET 4-6% or on-therapy AET 1-4%) and reflux episodes on impedance (normal < 80) were compared.

**Results:**

Of 889 HRM patients, 64 (7.6%) met IEM criteria and were compared to 466 controls. There were no significant differences in age, sex, dysphagia, reflux or globus symptoms between groups. Ambulatory pH testing was performed in 34 IEM patients (53.1%) and 297 controls (63.7%). Pathologic reflux and abnormal reflux on impedance was significantly more frequent in IEM vs controls (70.0% vs 41.8%, *p* = .012; 8.3% vs 66.2%, p = .022). Borderline reflux was not significantly different between groups (*p* = .41). Logistic regression confirmed pathologic reflux (OR 3.1, *p* = .006) as a predictor of IEM.

In 48 IEM patients with complete ESQ-30 data, dysphagia symptoms (ESQ-D) correlated weakly with % failed swallows (ρ = .292, *p* = .044); no other significant correlations were observed.

**Conclusions:**

Patients with IEM report a symptom burden similar to those with normal motility, but demonstrate significantly higher rates of pathologic reflux. Symptom severity may reflect esophageal dysfunction. These findings support the importance of reflux testing in IEM patients to detect occult GERD physiology.

**Funding Agencies:**

None

